# Multi-Element Characterization and Sustainability Assessment of Traditional Turkish Red Sugar Using Deep Eutectic Solvent Extraction and ICP-MS

**DOI:** 10.3390/foods15203719

**Published:** 2026-10-19

**Authors:** Nükte Topraksever

**Affiliations:** Faculty of Engineering and Natural Sciences, Chemical Engineering, Istanbul Health and Technology University, İstanbul 34275, Türkiye; nukte.topraksever@istun.edu.tr

**Keywords:** traditional Turkish red sugar, deep eutectic solvent, ICP-MS, multi-element analysis, food characterization, analytical sustainability

## Abstract

Traditional Turkish red sugar is a culturally significant food product; however, information on its elemental composition and the sustainability of analytical approaches used for its characterization remains limited. In this study, microwave-assisted extraction (MAE) and ultrasound-assisted deep eutectic solvent extraction (UA-DES-E) were comparatively evaluated for multi-element determination by ICP-MS. The analytical performance of both methods was validated in terms of linearity, limits of detection and quantification (LOD/LOQ), recovery, accuracy, and precision (RSD (%)). Complementary characterization was performed using FTIR and UV–Vis spectroscopy, while moisture content and antioxidant activity were also determined. In addition, the relationship between element concentrations and antioxidant capacity was statistically evaluated. Validation results showed linear correlation coefficients (R^2^) higher than 0.99. LOD values ranged between 0.172 and 2.288 µg kg^−1^, and recovery values were obtained between 96.2% and 105.1%. Relative standard deviation (RSD) values were below 10% for all elements. DPPH free radical scavenging activity ranged from 19.5% to 23.5%. The sustainability of the MAE and UA-DES-E methods was comparatively evaluated using NEMI, AGREE, ComplexGAPI, AGSA, BAGI, CACI, VIGI, and RAPI metrics. Overall, UA-DES-E provided a better balance between analytical performance and environmental sustainability. Based on five independent commercial samples, this study provides an exploratory analytical characterization of traditional Turkish red sugar while integrating multi-element analysis with a multidimensional assessment of analytical sustainability, applicability, innovation, and performance.

## 1. Introduction

Traditional food products hold significant importance in terms of preserving cultural heritage and maintaining sustainable dietary habits. These products are attracting attention today not only for their cultural value but also for their nutritional content and functional properties. Scientifically characterizing traditional foods is crucial for both protecting consumer health and establishing the quality and safety of these products [1,2].

Sugar-based traditional foods are primarily characterized by their high carbohydrate content but may also contain mineral elements originating from raw materials and processing. The elemental composition of foods is critical for determining nutritional values as well as assessing the presence of toxic elements. While macroelements such as calcium, magnesium, and potassium, and trace elements such as iron, copper, and manganese are essential for human metabolism, toxic elements such as lead, cadmium, and chromium can pose health risks even at low concentrations [3]. Therefore, multi-element determination in food products constitutes an important part of food safety studies.

Atomic absorption spectrometry (AAS) [4], atomic fluorescence spectrometry (AFS) [5], inductively coupled plasma optical emission spectrometry (ICP-OES) [6,7], and inductively coupled plasma mass spectrometry (ICP-MS) [8] are widely used in elemental analysis. Among these techniques, ICP-MS offers important advantages, including low detection limits and simultaneous multi-element determination, making it particularly suitable for elemental analysis in food matrices [9].

Sample preparation is a critical step in elemental analysis because it can directly influence analytical accuracy, precision, detection capability, and overall method performance. Conventional sample preparation procedures may require substantial reagent consumption, multiple processing steps and extended preparation times, thereby increasing waste generation and the environmental impact of the analytical procedure [10]. Consequently, increasing attention has been directed toward alternative sample preparation strategies that reduce reagent consumption, waste generation, and operator exposure while maintaining adequate analytical performance. In this context, miniaturized and alternative extraction approaches have gained increasing importance in green analytical chemistry [11].

Deep eutectic solvents (DESs) are considered environmentally friendly solvents due to their many beneficial properties. Instead of toxic and expensive solvents, the use of DESs as extraction solvents in microextraction methods has increased because they are easy to synthesize, inexpensive, and environmentally friendly. A DES is defined as a eutectic liquid mixture of two or more solid or liquid starting materials (a hydrogen bond acceptor (HBA) and a hydrogen bond donor (HBD)) capable of forming intermolecular hydrogen bonds and van der Waals interactions. The charge distribution resulting from hydrogen bonds between the HBA and HBD lowers the melting point of the resulting product compared to the melting points of each individual component [12]. The synthesis method for DESs involves mixing and heating under mild conditions until a homogeneous and transparent liquid is obtained. DES-based extraction approaches offer a more environmentally friendly alternative to conventional organic solvents [13].

Quantitative metrics should be used to assess the applicability, environmental sustainability, performance, and degree of innovation of analytical methods. A single criterion is generally insufficient; a multifaceted approach that considers the method holistically is required [14,15]. In recent years, with the development of environmentally friendly methods, more than 16 measurement tools have been developed to assess the degree of compliance with GAC concepts. Commonly used assessment tools include the National Environmental Methods Index (NEMI), the Green Analytical Procedure Index (GAPI), the Analytical Green Score Approach (AGSA), and the Analytical Green Calculator (AGREE) [16]. These criteria take into account all aspects of the method to determine the “green index” of the analytical procedure. White Analytical Chemistry (WAC) offers a holistic approach that encompasses both environmental, analytical, and practical aspects [17]. As an alternative to the 12 GAC principles, WAC has criteria for environmental impact, analytical aspects, and feasibility, which are represented by green, red, and blue, respectively. However, the Violet Innovation Grade Index (VIGI), which reflects the method’s innovativeness, is a new metric. VIGI serves as a complementary measure to the existing green criteria, in addition to the feasibility parameters [18].

In this study, sensitive and reliable determination of Ca, Cd, Cr, Cu, Fe, K, Mg, Mn, and Pb elements was performed using microwave-assisted extraction (MAE) and ultrasound-assisted deep eutectic solvent extraction (UA-DES-E) approaches in traditional Turkish red sugar via ICP-MS. The aim was to comprehensively evaluate the developed methods not only in terms of analytical performance parameters (accuracy, sensitivity, recovery, and detection limits) but also in terms of environmental sustainability. Accordingly, the methods were systematically and comparatively analyzed using multiple metrics based on different principles such as NEMI, AGREE, ComplexGAPI, AGSA, BAGI, CACI, VIGI, and RAPI. Thus, instead of being limited to a single metric, parameters such as reagent consumption, energy requirement, waste generation, operator safety, innovation, and method complexity were addressed with a holistic approach. Furthermore, FTIR and UV spectrophotometric analyses were performed to reveal the matrix properties of the samples, and antioxidant activity and moisture content were determined. The potential relationships between elemental composition and antioxidant capacity were statistically evaluated to investigate the contribution of mineral content to functional properties. Therefore, this study provides a comprehensive chemical characterization of traditional Turkish red sugar by integrating elemental composition, spectroscopic properties, antioxidant activity, and sustainability assessment within a single analytical framework.

To the best of our knowledge, no previous study has combined this DES-based extraction, ICP-MS multi-element analysis, spectroscopic characterization (FTIR and UV-Vis), antioxidant evaluation, and multidimensional sustainability metrics for traditional Turkish red sugar.

## 2. Materials and Methods

### 2.1. Chemicals and Reagents

The choline chloride (ChCl), lactic acid (LA), sodium hydroxide, hydrogen peroxide, nitric acid and hydrochloric acid used in the experiments were purchased from Merck (Darmstadt, Germany). 2,2-Diphenyl-1-picrylhydrazyl (DPPH) and methanol were purchased from Sigma-Aldrich (St. Louis, MO, USA). Furthermore, ultrapure water (resistivity of 18.2 MΩ·cm) obtained from a Human Power I water purification system (Human Corporation, Seoul, Republic of Korea) was used in all experiments. All solutions were prepared fresh daily, and samples were stored in a refrigerator at +4 °C until analysis.

### 2.2. Instrumentation

Moisture content was determined using a halogen moisture analyzer (MA 50.R, Radwag, Radom, Poland). A vortex mixer (F202A0173, Velp Scientifica, Usmate, Italy) and an ultrasonic bath (WF-UD6, Weightlab Instruments, Istanbul, Türkiye) were used during sample preparation. Phase separation was performed using a centrifuge (NF400, Nüve, Ankara, Türkiye), and pH measurements were carried out using a pH meter (HI2002-01, Hanna Instruments, Woonsocket, RI, USA). FTIR measurements were performed using a Bruker Alpha FTIR spectrometer (Bruker, Ettlingen, Germany), and spectra were acquired and evaluated using OPUS software (Bruker Optik GmbH, Ettlingen, Germany; https://www.bruker.com, accessed on 15 October 2026). UV–Vis measurements were carried out using an Agilent 8453 spectrophotometer (G1103A, Agilent Technologies, Santa Clara, CA, USA) operated with UV-Visible ChemStation software (Agilent Technologies, Santa Clara, CA, USA; https://www.agilent.com, accessed on 15 October 2026). Elemental analyses were performed using an Agilent 7700 series inductively coupled plasma mass spectrometer (ICP-MS) (Agilent Technologies, Santa Clara, CA, USA), and data were processed using MassHunter software (version B.01.03, Agilent Technologies, Santa Clara, CA, USA).

### 2.3. Sample Collection

Five independent commercially available traditional Turkish red sugar samples were purchased from different local herbal retailers in Tekirdağ, Türkiye, and coded as S1–S5. The traditional Turkish red sugar samples were evaluated for moisture content, FTIR-based structural functional group characterization, UV spectral analysis, and elemental content via ICP-MS. Each analytical measurement was performed in triplicate, and the results are expressed as mean ± standard deviation (SD).

### 2.4. Synthesis and Characterization of DES

**Figure 1:**
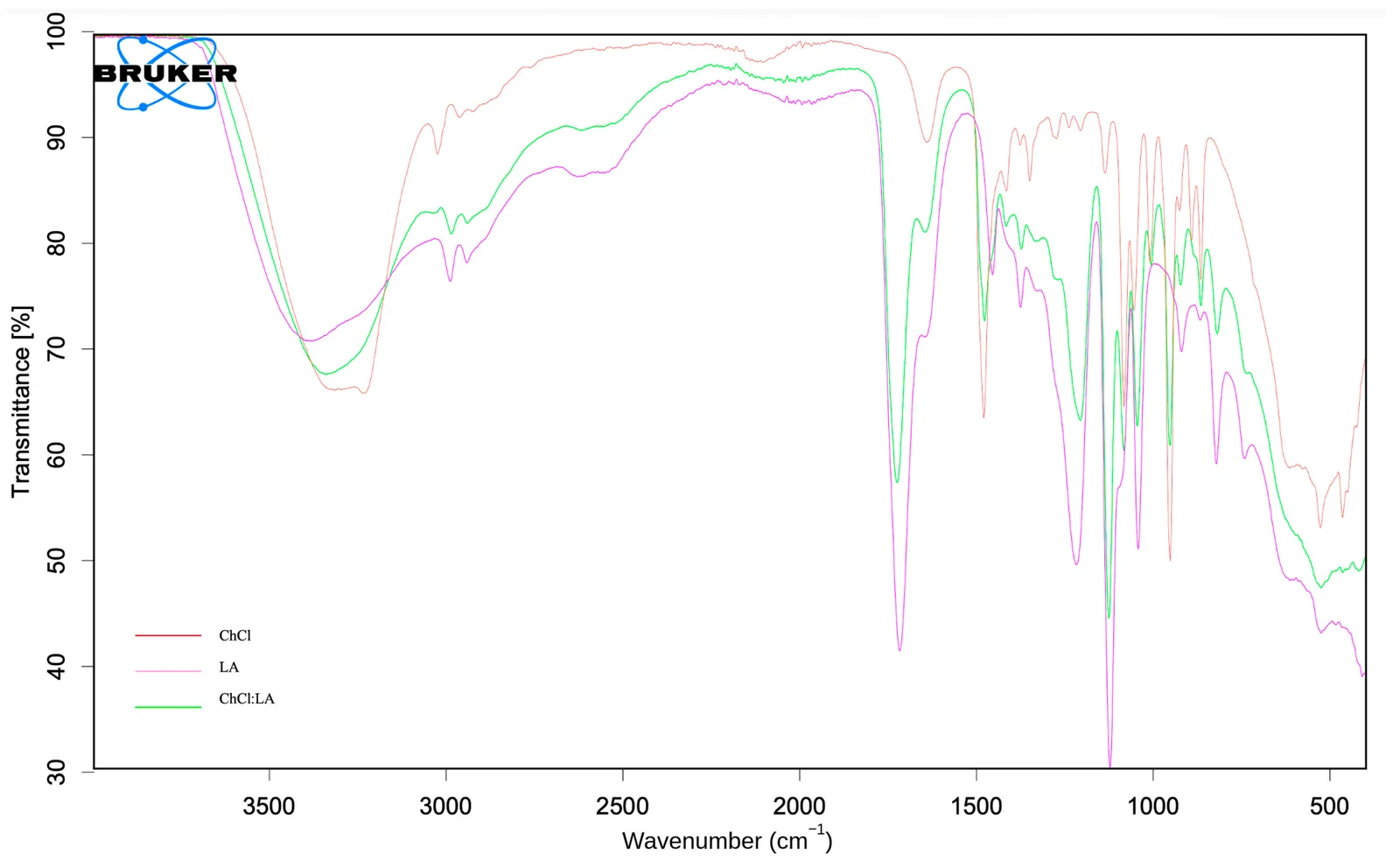
FTIR spectra of choline chloride (ChCl), lactic acid (LA), and the ChCl:LA deep eutectic solvent (DES).

A two-component DES solution consisting of choline chloride (ChCl) and lactic acid (LA) (in a 1:1 molar ratio) was stirred on a magnetic stirrer (JSHS-18A, JS Research Inc., Gongju, Republic of Korea) at 80 °C and 400 rpm until a clear liquid solution was obtained. The synthesized DES was stored in dark-colored glass bottles at room temperature. The resulting DES was characterized by ATR-FTIR spectroscopy. The synthesized DES was confirmed to consist of hydrogen bonds between the carboxyl and hydroxyl groups of the starting components, as well as intermolecular interactions [19,20]. ATR-FTIR spectra confirm the DES synthesis (Figure 1).

The ChCl spectrum showed characteristic absorption bands corresponding to amino and hydroxyl groups at 1200–800 cm^−1^ and 3250 cm^−1^, -C-H stretching vibration peak at 3000 cm^−1^, and a -C-H bending vibration peak at 1501 cm^−1^ [21,22]. The CH_2_ bending at 1501 cm^−1^ is a distinct group detected in all ChCl-based DESs.

The LA spectrum is characterized by a broad peak at 3500–3000 cm^−1^ and 2985 cm^−1^ corresponding to the -O-H and -C-H stretching vibrations. Additionally, the strong band at 1700 cm^−1^ belongs to the C=O stretching vibration [21].

After DES formation, a shift to lower wavenumbers occurs in the LA -O-H stretching vibration. Furthermore, the intensity of the C=O absorption band at 1700 cm^−1^ has decreased and shifted.

The disappearance of the vibration band at 1349 cm^−1^ in ChCl-based DESs has been observed [23,24].

### 2.5. Extraction Procedures

#### 2.5.1. MAE

0.5 g of sample was weighed, and 7 mL of 65% nitric acid (HNO_3_) and 1 mL of 30% hydrogen peroxide (H_2_O_2_) were added to the samples. The samples were transferred into Teflon vessels and subjected to microwave-assisted digestion using a Milestone START D microwave digestion system (Milestone Srl, Sorisole, Italy), equipped with a high-pressure digestion rotor with a maximum pressure capacity of 100 bar.

The temperature was raised to 200 °C in 15 min, and then the dissolution process was carried out at 200 °C for an additional 15 min at a microwave power of 120 W (the samples were digested until a clear solution without visible sediment was obtained). Then, the volume was diluted to 50 mL with ultrapure water and stored at +4 °C. The same procedures were performed for the reagent blank as well. Ultrapure water was used in all experiments, and the experiments were repeated three times. Samples were stored in the refrigerator at +4 °C until analysis [25].

#### 2.5.2. UA-DES-E

A 0.25 g portion of each sample was weighed and mixed with 5 mL of an extraction medium consisting of the previously prepared ChCl:LA DES (1:1 molar ratio) and water at a DES:water ratio of 80:20 (*w*/*w*) using a vortex mixer. The mixture was incubated in a 50 °C ultrasonic bath for 1 h. It was then centrifuged at 4500 rpm for 5 min to remove undissolved solid particles and filtered. Finally, 1 mL of sample was taken and diluted to 10 mL with 1% (*w*/*w*) HNO_3_. The same procedures were applied to the blank sample. Ultrapure water was used in all experiments, and the experiments were repeated three times. Samples were stored in the refrigerator at +4 °C until analysis [26].

### 2.6. Antioxidant Properties

The sample solutions used for the DPPH assay were prepared separately from those used for ICP-MS analysis. Briefly, 1.0 g of each sugar sample was dissolved in 10 mL of ultrapure water, and the resulting aqueous sample solution was used for the DPPH assay. The antioxidant activity of the extracts was evaluated using DPPH spectrophotometric measurement at 517 nm. The analysis involved diluting 0.0079 g of DPPH in 40 mL of an 80% (*v*/*v*) methanol solution. Following the addition of 3000 µL of diluted DPPH solution and 600 µL of methanol (80%, *v*/*v*) to 100 µL of the sample solution, the mixture was incubated in the dark for 30 min. The DPPH radical scavenging activity (%) was determined using the following equation:

DPPH scavenging effect (%) = [(A_control_ − A_sample_)/A_control_] × 100

A_control_ denotes the absorbance of the diluted DPPH solution, while A_sample_ signifies the absorbance of the sample [27].

### 2.7. ICP-MS Analysis

After extraction, each sample was fed into the ICP-MS via a crossflow nebulizer, sprayer and autosampler, and then Ca, Mg, K, Mn, Cu, Fe, Cr, Cd, and Pb (macro, micro, and toxic) were determined. The operating parameters of the ICP-MS spectrometer are presented in Table 1.

### 2.8. Physicochemical and Spectroscopic Analysis

The moisture content of the sugar samples was determined using a halogen moisture analyzer. Approximately 3 g of the sugar sample was weighed and spread evenly in the sample pan after being broken into small pieces. Drying was continued at 103 °C until the weight stabilized (automatic shut-off mode). All measurements were performed in triplicate [28].

Fourier Transform Infrared Spectroscopy (FTIR) and UV-Vis spectrophotometric analyses were performed to structurally characterize the samples and determine their functional group distribution. Spectra were recorded in the appropriate wavelength range, and hydroxyl (O–H), aliphatic C–H, carbonyl (C=O), and C–O–C stretching vibrational bands were evaluated. UV-Vis spectrophotometric analyses were performed to examine the absorbance properties of the samples at specific wavelengths. Measurements were performed in a suitable solvent environment at the specified wavelength ranges, and the obtained spectral data were used to support the structural characterization studies.

### 2.9. Method Validation

The analytical performance of the developed MAE- and UA-DES-E-based sample preparation methods in element determination by ICP-MS was evaluated in accordance with international validation criteria [29]. The accuracy, precision, linearity, limit of detection (LOD), and limit of quantification (LOQ) parameters of the method were determined. Linearity was evaluated using four calibration levels prepared over the corresponding concentration range for each element, and the coefficients of determination (R^2^) were calculated from the resulting calibration curves. Calibration standards covering concentration ranges of 10–100 µg/L (ppb) and 0.5–5 mg/L (ppm) were available for trace-level and higher-concentration elemental determinations, respectively. LOD and LOQ were calculated according to the standard deviation–slope approach using the equations LOD = 3.3 σ/m and LOQ = 10 σ/m, where σ represents the standard deviation obtained from ten independent blank measurements (*n* = 10), and m represents the slope of the corresponding calibration curve. Procedural blanks containing all reagents used in the corresponding sample-preparation procedure, but without the sample, were processed in parallel with the samples. The elemental background measured in these blanks was taken into account during data processing, and the reported sample concentrations were blank-corrected with the corresponding dilution factors applied. The repeatability (intra-day precision) and inter-day precision of the method were calculated as percentage relative standard deviation (%RSD) using consecutive measurements performed on the same sample. As part of the accuracy evaluation, recovery studies were carried out by adding known amounts of element standard (spike) to the samples, and percentage recovery values were determined. Recovery experiments were performed by spiking 1 mL of sample with 1 mL of a standard solution. A 25 µg/L (ppb) standard solution was used for trace-level determinations, whereas a 0.5 mg/L (ppm) standard solution was used for elements evaluated at higher concentration levels. The recovery values obtained from these experiments ranged from 96.2% to 105.1%, supporting the accuracy of the analytical procedure at the evaluated spiking levels. A matrix-matched certified reference material (CRM) with certified values for the target elements was not used in the present study. Therefore, analytical accuracy was evaluated by spike-recovery experiments performed directly in the analyzed sample matrix (Table 2). Statistical analyses (paired *t*-test and Pearson correlation) were performed using Microsoft Excel (Microsoft 365; Microsoft Corporation, Redmond, WA, USA). The correlation heatmap (Figure 4) was generated from the Excel-calculated correlation coefficients using ChatGPT (GPT-5.6, OpenAI, San Francisco, CA, USA).

### 2.10. Four-Dimensional Methodological Assessment: Integrating Sustainability, Practicality, Innovation, and Performance

This section describes the criteria used to evaluate sample preparation methods employing choline chloride:lactic acid-based DES and acid-assisted microwave digestion of Ca, K, Mg, Cu, Fe, Mn, Cd, Cr, and Pb from sugar samples. Calculations were performed considering energy consumption, waste generation, and extraction data obtained from ICP-MS instruments to assess whether the methods are environmentally friendly, feasible, innovative, and possess good analytical performance. The Analytical GREEnness Metric (AGREE), National Environmental Methods Index (NEMI), Complex Green Analytical Procedure Index (ComplexGAPI), Analytical Green Star Area (AGSA), Blue Applicability Grade Index (BAGI), Click Analytical Chemistry Index (CACI), Violet Innovation Grade Index (VIGI), and Red Analytical Performance Index (RAPI) metrics were compared for both methods.

The eight assessment metrics were selected to provide a multidimensional evaluation of the analytical procedures from complementary perspectives. NEMI, AGREE, ComplexGAPI, and AGSA characterize environmental sustainability using different assessment frameworks, while BAGI evaluates practical applicability and CACI addresses operational and methodological aspects. VIGI provides an innovation-oriented evaluation, whereas RAPI focuses on analytical performance. The integration of these metrics therefore enables the MAE and UA-DES-E procedures to be characterized across four key dimensions—environmental sustainability, practical applicability, methodological innovation, and analytical performance—providing a structured and comprehensive assessment of the overall analytical workflow.

#### 2.10.1. Environmental Sustainability (Greenness)

NEMI is one of the oldest tools for assessing the environmental friendliness of an analytical method developed by the Multi-Disciplinary Methods and Data Comparability Board (MDCB). NEMI appears in green when the relevant criteria are met within a circle divided into four sections. These criteria include whether the reagents are listed as persistent, bioaccumulative, and toxic (PBT), whether they are classified as corrosive or hazardous waste, and whether the waste generated by the procedure is less than 50 g [30]. NEMI provides a quick visual assessment of the method’s greenness but offers limited quantitative assessment [31].

AGREE is a metric used to numerically assess the “greenness” of a procedure. It is based on the twelve principles of green analytical chemistry. The “greenness” score is displayed within a clock-face-like circle, and colors representing each principle are visually depicted along the circumference of the circle (from red to green). The method’s level of environmental friendliness is displayed numerically at the center of the circle. Scores range from 0 to 1. Scores closer to 1 indicate a higher level of environmental friendliness, while scores closer to 0 indicate a lower level [32].

GAPI was proposed by Płotka-Wasylka [15]. The environmental friendliness of the various stages of analytical methods can be assessed using the five pentagrams in the GAPI pictogram. To indicate the degree to which the different stages of the analytical method are environmentally friendly, a color scale consisting of three different levels is applied in the GAPI pictogram (green, yellow, and red colors indicate that the analytical method’s impact on the environment and human health is low, moderate, and high, respectively). Sections that comply with green metric principles are marked in green. If the relevant criterion is not met, the section is marked yellow or red. This indicates that the method is hazardous, not environmentally friendly, or poses a risk to the analyst. This metric is a semi-quantitative assessment tool [30]. In 2021, J. Płotka-Wasylka and W. Wojnowski introduced the complex GAPI, an extension of the original GAPI tool [33]. The complex GAPI includes an additional hexagon in addition to the pictogram found in the original GAPI and assesses whether the procedures prior to analysis and sample pretreatment are also environmentally friendly [34]. This hexagon covers various topics, including yield, reaction conditions, compliance with green economy principles, reagents and solvents, instrumentation, processing, purification of final products, waste management, and environmental factors. The E-factor is a metric used to assess the environmental impact of a chemical process. It is calculated as the ratio of the total mass of waste produced to the mass of the product obtained [35]. A low E factor indicates that the amount of product produced exceeds the amount of waste generated, and that the procedure and process are environmentally friendly [31].

The AGSA metric evaluates the degree to which an analytical method is environmentally friendly by assigning a score. Based on the 12 principles of green analytical chemistry, each principle is assessed through specific questions with three-level response options. Higher scores indicate greater sustainability and support strategies such as minimizing the sample preparation process, low energy consumption, the use of non-hazardous reagents, and minimizing waste [36].

#### 2.10.2. Operational Practicality (Applicability)

BAGI is used to evaluate the applicability of an analytical method. A numerical value between 25 and 100 is determined, and the higher the value, the more applicable the method is. BAGI is based on the 10 characteristics of White Analytical Chemistry. The criteria are scored on a scale of 2.5 to 10 points. These characteristics are as follows: 1–3 correspond to analytical determination steps; 4–5 correspond to sample preparation steps; and characteristics 6–10 correspond to both analytical determination and sample preparation steps. Parameters 1–5 are located inside the pictogram, while parameters 6–10 are located outside the pictogram. BAGI uses a color-coded system: dark blue for high scores and white for low scores [7,37].

The key parameters include preparation, sample volume, applicability, portability, usability, accuracy, and automation. The CACI pictogram’s color code indicates how the method performs for each parameter. A colored pictogram indicates excellent performance; a gray pictogram indicates moderate performance; and a black pictogram indicates poor performance or failure to meet the desired criteria [38].

#### 2.10.3. Methodological Innovation (Innovation)

VIGI is a new assessment metric used to assess the innovativeness of analytical methods. It is based on 10 questions related to the analytical method and the ratings given to these questions. For ease of evaluation, three different score values are used: 0, 5, and 10. Each score is represented by shades of purple—dark purple, light purple, and white—which indicate the level of innovation from high to low. The result of the VIGI application is a purple, decagon-shaped pictogram. The VIGI pictogram is evaluated based on two key parameters. First, the darker the shade of purple, the more innovative the method is. Second, the number on the pictogram represents the analytical method’s overall score on a 0–100 scale; the higher this number, the more innovative the method is [18].

#### 2.10.4. Analytical Performance (Reliability)

RAPI is an analytical performance assessment metric supported by Python-based software (https://rapi-index.anvil.app, accessed on 15 October 2026). RAPI evaluation parameters are based on ICH recommendations, generally accepted criteria, and good laboratory practices. These parameters include repeatability, reproducibility, accuracy, recovery, LOQ, working range, linearity, robustness, and selectivity. The RAPI scoring system consists of five levels ranging from 0 (worst result) to 2.5, 5.0, 7.5, and 10 (best result). A value of 0 is assigned to a parameter that has not been tested. The sum of all scores in the 0–100 range is located at the center of the pictogram. The better the overall performance of the method, the higher this value is. It is displayed on the pictogram as a scale ranging from light red to dark red [39].

## 3. Results

### 3.1. Moisture Content Analysis of Sugar Samples

The moisture content of the sugar samples is given in Table 3. The moisture range of the samples is 2.09–4.69%. The moisture content, which is also a quality parameter for hard candies, should be in the range of 2–3%. Low moisture content improves the microbial, physical, and flavor stability of the product by providing low water activity. Water content above 5% disrupts the glassy structure and results in products with a deteriorated texture. Low moisture content is a necessary parameter for a long shelf life [40].

### 3.2. Spectroscopic Profile (FTIR & UV-Vis)

**Figure 2:**
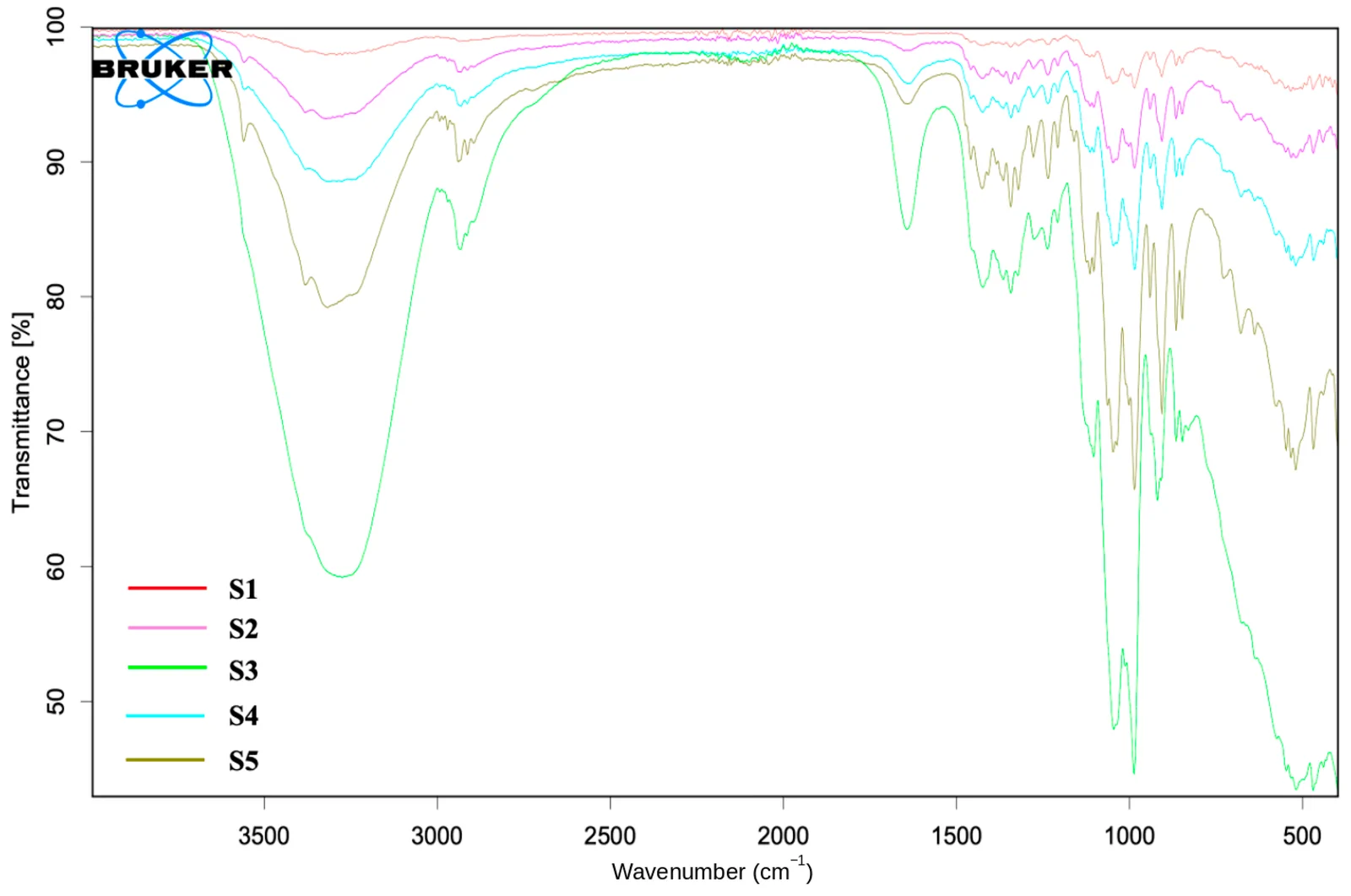
Overlaid FTIR spectra of the analyzed samples.

**Figure 3:**
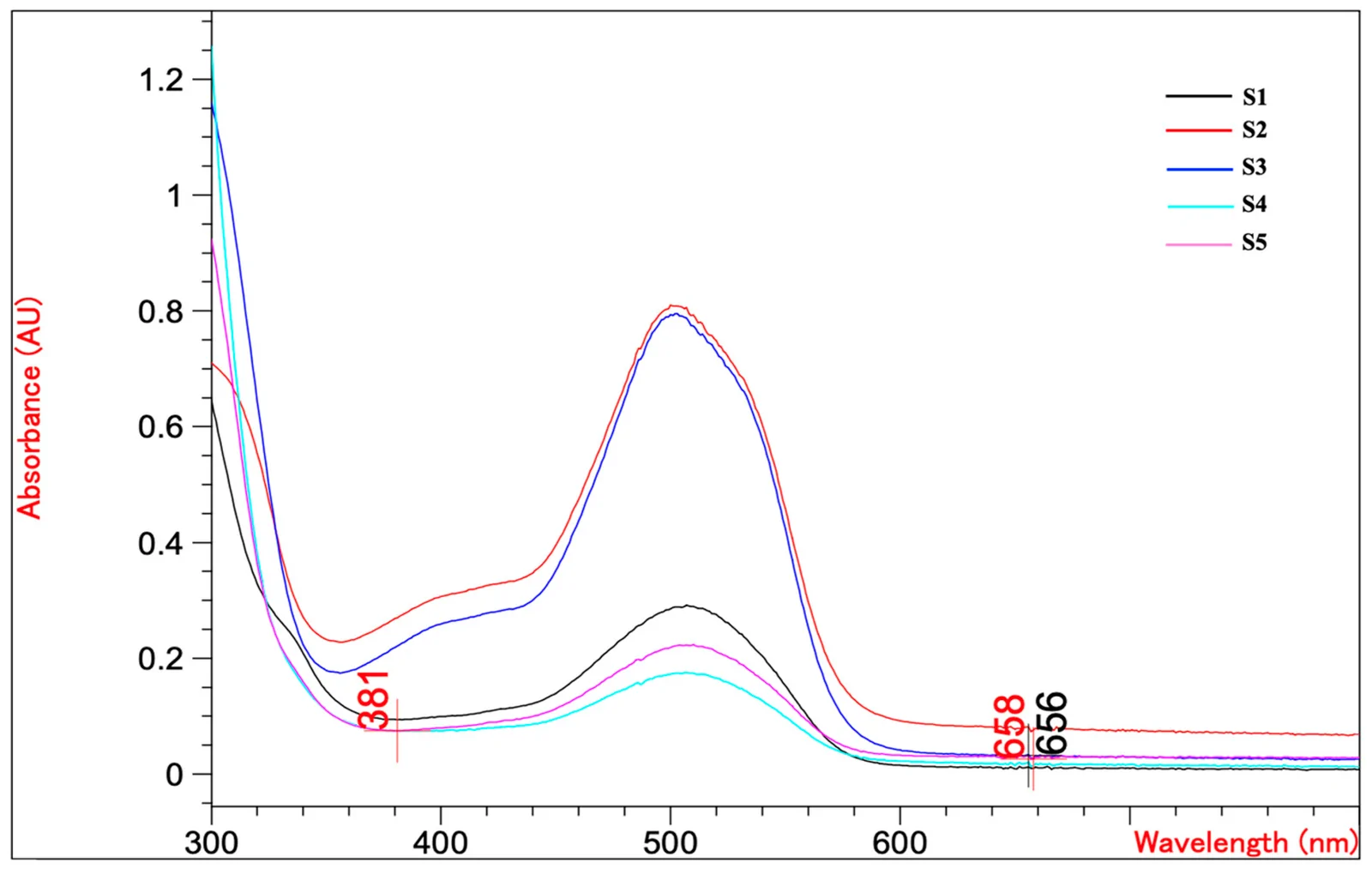
Overlaid UV spectra of the analyzed samples.

When FTIR spectra of sugar samples were obtained, characteristic absorption bands were observed. Furthermore, the spectra of matching substances were compared with the instrument library and are given in Supporting Information. In the samples, characteristic absorption bands were consistent with functional groups commonly associated with cinnamon, ginger and clove-containing formulations. The main absorption bands were observed at Hydroxyl (-OH) stretching at 3300 cm^−1^, C-H stretching at 2900 cm^−1^, carbonyl (C=O) stretching at 1600 cm^−1^, and (C-O) and (C-O-C) bonds at 1000–1150 cm^−1^. Bands observed in the 1600–1640 cm^−1^ and 1490–1500 cm^−1^ regions may be consistent with structural features associated with azo-type colorants; however, FTIR analysis alone does not provide definitive identification of a specific food colorant. In UV–Vis spectrophotometric measurements, an absorption maximum at approximately 504 nm was observed. This spectral feature is consistent with the possible presence of a red chromophore; however, definitive identification of Allura Red AC (E129) would require confirmation using a selective analytical technique (Figure 2 and Figure 3; Figure S1 (Supplementary Information—SI)).

### 3.3. Determination of Antioxidant Activity

The DPPH scavenging effects of five different samples were calculated as percentages, and the results are given in Table 4.

### 3.4. Elemental Composition and ICP-MS Findings

Extraction of Ca, Cd, Cr, Cu, Fe, K, Mg, Mn, and Pb in sugar samples was performed using MAE and UA-DES-E methods, and their determination was carried out using ICP-MS; the results are given in Table 5.

The elemental concentrations determined in the present study were compared with previously reported data for sugar, confectionery, and other sugar-based food matrices [4,41,42,43,44,45]. Overall, the concentrations observed in traditional Turkish red sugar fell within the broad concentration ranges reported for related products, although considerable differences among food matrices have been documented in the literature. For example, previous studies have reported markedly variable Ca, Mg, and K concentrations among sugar, chocolate, jelly candy, hard candy, and other sugar-based products, while similarly broad variations have been observed for Fe, Cu, and Mn [4,41,42,43,44]. Such variability indicates that elemental composition cannot be interpreted independently of the specific food matrix and product formulation. The present samples likewise exhibited substantial variation in their elemental profiles, particularly for Ca, Mg, Fe, Mn, Cr, and Pb. These differences may be associated with variations in the raw materials used in traditional red sugar formulations, as well as differences in processing and production conditions. For the potentially toxic elements, Cd remained below the LOQ in all samples, whereas Cr and particularly Pb showed greater sample-dependent variation. Previous studies on sugar and confectionery products have also reported heterogeneous findings for these elements, ranging from non-detectable levels to measurable concentrations depending on the product and analytical procedure [41,42,45]. Therefore, comparison with previous studies provides useful analytical context, but differences in matrix composition, sample preparation, and instrumental methodology should be considered when interpreting absolute concentration differences across studies.

The differences observed between MAE and UA-DES-E should also be considered in relation to the fundamentally different principles of the two sample-preparation approaches. MAE employs concentrated HNO_3_/H_2_O_2_ under elevated temperature and microwave-assisted conditions, promoting extensive decomposition of the sugar-rich matrix and release of the elements into the analytical solution. In contrast, UA-DES-E relies on the transfer of elements from the sample matrix into a ChCl–LA-based extraction medium under considerably milder conditions. Therefore, its extraction behavior may be more strongly influenced by the accessibility and chemical environment of individual elements within the matrix, as well as their interactions with the DES components. The applicability of choline chloride-based DESs combined with ultrasound for the extraction of chemical elements from complex matrices has also been demonstrated previously [26]. Ultrasound-assisted extraction can enhance contact between the solid sample and the extraction medium and facilitate mass transfer. Accordingly, the element-dependent differences observed between MAE and UA-DES-E in the present study may reflect the distinct extraction mechanisms of the two procedures and matrix-dependent extraction behavior, rather than a uniform response of all elements to the two sample-preparation approaches.

The relatively high Pb concentrations observed in some samples may reflect not only the elemental composition of the raw materials but also differences associated with traditional production and processing practices. Food-contact materials and processing equipment may contribute to metal contamination during food preparation, particularly when certain traditional cookware materials are used. However, since detailed information on the production conditions and utensils used for the analyzed commercial samples was not available, the specific origin of Pb cannot be established in the present study. Notably, Pb showed pronounced sample-specific differences between the two extraction procedures. For example, in S3, the Pb concentration obtained by UA-DES-E (844.9 µg/kg) was substantially higher than that obtained by MAE (259.6 µg/kg). Considering the distinct extraction principles of MAE and UA-DES-E, this discrepancy may reflect matrix-dependent differences in Pb extraction and recovery. Importantly, the absence of a statistically significant difference in the paired *t*-test should not be interpreted as evidence of agreement between the two procedures, particularly given the limited number of independent samples. These findings therefore highlight the importance of monitoring Pb in traditionally produced foods and considering production-related factors in future studies. A comprehensive dietary risk assessment would additionally require product-specific consumption data, which was beyond the scope of the present analytical characterization.

In the statistical analysis, a paired *t*-test was applied using the average concentration values obtained for each element in the five samples (S1–S5) to compare the results obtained by the MAE and UA-DES-E procedures. All calculations were performed at a 95% confidence level (α = 0.05). According to the statistical findings (Table 5), no statistically significant difference was detected between the two extraction procedures for Ca, Cr, Cu, Fe, K, Mg, and Pb (*p* > 0.05), whereas a statistically significant difference was observed for Mn (*p* < 0.05). Considering the limited number of independent samples (*n* = 5), these results are interpreted only as a statistical comparison of the concentration values obtained by the two extraction procedures and not as evidence of method agreement, equivalence, or interchangeability.

### 3.5. Evaluation of the Relationship Between Elemental Composition and DPPH Antioxidant Activity

**Figure 4:**
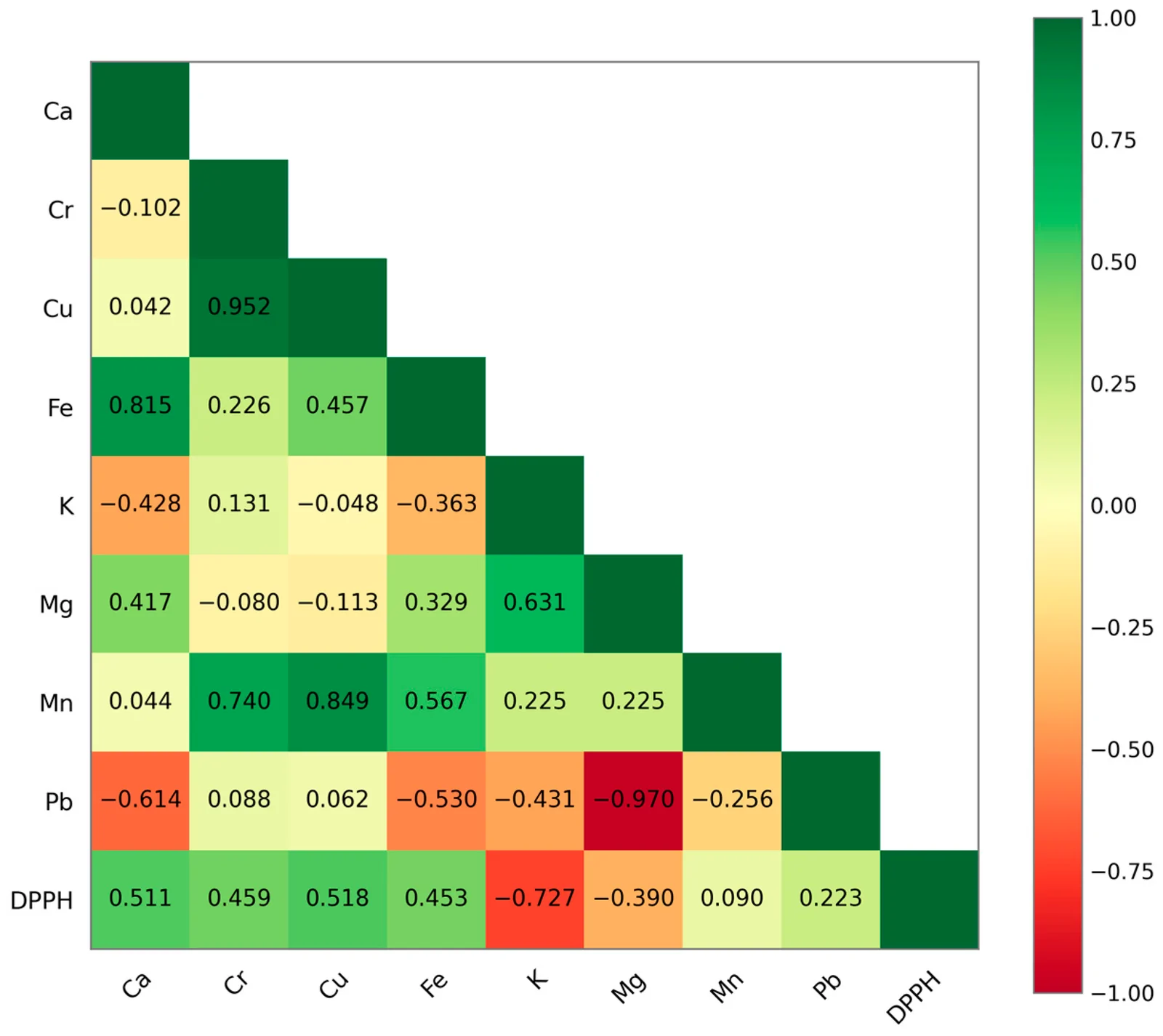
Heatmap of Pearson correlation coefficients showing the relationships between elemental concentrations and DPPH radical scavenging activity.

The relationships between the elemental composition of the samples and DPPH radical scavenging activity were evaluated using Pearson correlation analysis, and the results are presented in Figure 4. Given the limited number of independent commercial samples (*n* = 5), the correlation analysis was considered exploratory and was used to identify potential patterns rather than to establish statistically robust or causal relationships. In general, positive correlations were observed between DPPH activity and Ca, Cu, and Fe, while negative correlations were found with K and Mg. The DPPH test is a fast and sensitive radical scavenging method widely used in the investigation of antioxidant capacity [46]. Studies show that DPPH radical scavenging capacity can be altered, especially by phenolic compounds and metal-ion interactions; for example, direct chemical interactions have been reported between transition metals such as Cu and Fe and antioxidant reaction mechanisms [47].

Moderate positive correlation coefficients were observed for Ca–DPPH, Cu–DPPH, and Fe–DPPH; however, these trends should be interpreted cautiously because of the limited sample size [46]. Furthermore, weak correlations were observed between Mn and Pb concentrations and DPPH activity. When looking at inter-element correlations, positive inter-element correlations, including Cr–Cu and Cu–Mn, were also observed; however, source attribution cannot be established from correlation analysis alone. These findings highlight the importance of considering the behavior of elements within a complex matrix in conjunction with antioxidant measurements [46,47].

### 3.6. Multi-Dimensional Evaluation of the Analytical Strategies: Sustainability, Practicality, Innovation, and Performance

Table 6 shows the evaluation of the MAE and UA-DES-E methods according to NEMI. As shown in Table 6, the UA-DES-E method is green in four areas, while the HNO_3_ and H_2_O_2_ used in the MAE method are hazardous and toxic, and the amount of waste produced does not qualify as green.

The AGREE assessment of the MAE and UA-DES-E methods was performed using an analytical greenness calculator (https://agree-index.anvil.app, accessed on 15 October 2026) [14]. The AGREE results for the UA-DES-E and MAE methods were found to be 0.71 and 0.35, respectively, and are shown in Table 6. According to Table 6, the UA-DES-E method received a value close to 1 based on the principles of low sample quantity, no need for derivatization, the ability to determine multiple analytes simultaneously, the use of renewable reagents, the absence of toxic reagents, and no threat to operator safety. In contrast, the MAE method received values close to 1 only for the principles of low sample quantity, no need for derivatization, and enabling the determination of multiple analytes, but received low values for other green chemistry principles, as shown in red.

Complex GAPI assessment indicated that UA-DES-E (left) is environmentally more favorable than MAE (right), with fewer red zones and improved sustainability in sample preparation and analytical steps (Table 6) (https://fotouhmansour.github.io/ComplexMoGAPI/, accessed on 15 October 2026) [48].

AGSA scores for the methods were calculated online at https://fotouhmansour.github.io/AGSA/ (accessed on 15 October 2026) [36]. Table 6 shows the AGSA scores for the MAE and UA-DES-E methods. The scores are 70.83 and 58.33 for UA-DES-E and MAE, respectively. UA-DES-E received a greener score due to the use of non-toxic solvents, being a safe method for the operator, not requiring derivatization, having a small sample size, and involving fewer pretreatment steps. MAE received a lower green score due to the use of concentrated acid, the pretreatment step, microwave energy consumption, toxic waste, and risky equipment for the operator.

As shown in Table 6, both the MAE and UA-DES-E methods have an applicable BAGI score. The BAGI scores for MAE and UA-DES-E are 65 and 72.5, respectively (https://bagi-index.anvil.app, accessed on 15 October 2026) [49]. Furthermore, Table 6 provides the 10 assessment criteria of the BAGI method. In this context, the criterion that the reagents used in the method are commonly available reagents in the commercial market is high for MAE, while UA-DES-E has a lower score because DES is a specially designed solvent. UA-DES-E is more applicable in terms of criteria such as fewer sample preparation steps, not including additional steps such as preconcentration, and a high number of samples prepared per hour. MAE and UA-DES-E have the same BAGI scores in terms of quantitative application and sample quantity.

Applications with a CACI score between 50% and 75% are considered acceptable in terms of practicality. A score of 75% or higher indicates that the method is quite practical, while a score below 50% indicates that the method’s practical usability is insufficient. Table 6 presents a comparison of CACI scores of the MAE and UA-DES-E methods, which was made according to the CACI software (https://bit.ly/CACI2025, accessed on 15 October 2026) [38]. The CACI scores were 78 for MAE and 72 for UA-DES-E, indicating that MAE was classified as highly practical, while UA-DES-E showed acceptable practicality.

According to the VIGI tool (https://bit.ly/VIGItool, accessed on 15 October 2026) mentioned in the study by [18], the UA-DES-E and MAE methods were compared, and the results are given in Table 6. The VIGI value of the MAE method is 25, while that of the UA-DES-E method is 60. As can be seen from the results, the UA-DES-E method is an innovative method due to its reactivity and waste, fewer steps, novel design, use of biodegradable solvents, and interdisciplinary nature. MAE received a low VIGI value due to reasons such as the use of traditional strong acids, higher waste generation, and lack of miniaturization.

**Figure 5:**
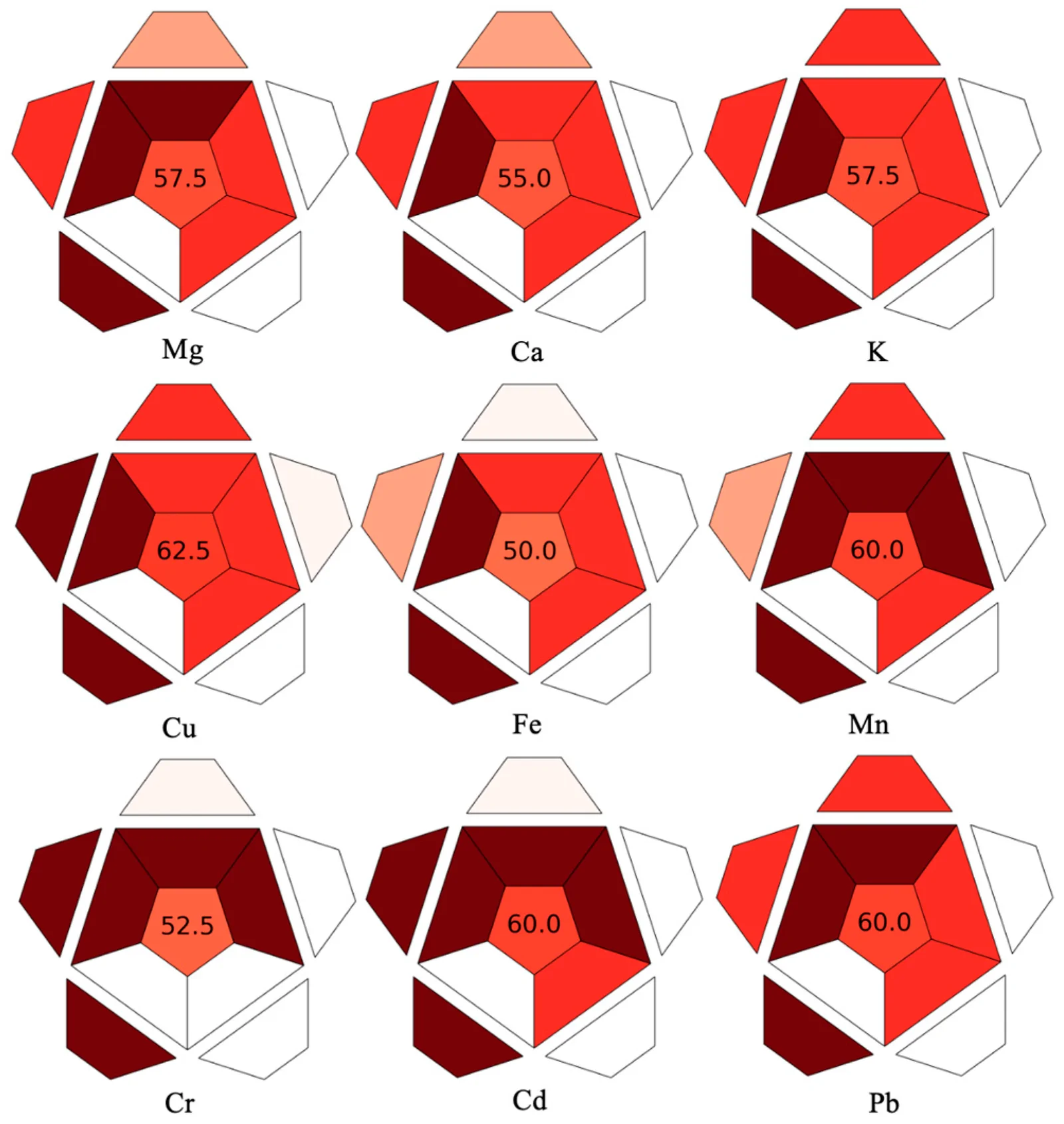
Red Analytical Performance Index (RAPI) evaluation of nine elements.

Figure 5 shows the RAPI evaluations for the determination of Ca, Cd, Cr, Cu, Fe, K, Mg, Mn, and Pb elements in sugar samples using ICP-MS. The RAPI values for each element range from 50 to 62.5. This indicates that the calculated parameters show that the analytical performance of the method is acceptable (https://mostwiedzy.pl/en/justyna-plotka-wasylka,647762-1/rapi, accessed on 15 October 2026) [39].

## 4. Discussion

The results of this study showed that both MAE and UA-DES-E provided satisfactory analytical performance for the multi-element determination of traditional Turkish red sugar by ICP-MS. The recovery and precision values obtained for both methods supported their applicability under the investigated conditions. However, the two extraction approaches differed in their operational characteristics. While MAE relies on concentrated acids, elevated temperature, and microwave energy, UA-DES-E uses a ChCl–lactic acid-based deep eutectic solvent (DES) and ultrasound-assisted extraction (UAE) under milder conditions. The combined sustainability assessment using NEMI, AGREE, ComplexGAPI, AGSA, BAGI, CACI, VIGI, and RAPI enabled these differences to be evaluated from complementary perspectives rather than relying on a single metric.

Variations in elemental composition, antioxidant activity, moisture content, and spectroscopic profiles were observed among the commercial samples. However, the correlations between elemental concentrations and antioxidant activity should be interpreted as exploratory because only five independent samples were investigated. In addition, FTIR and UV–Vis findings alone cannot provide definitive identification of a specific food colorant. Accordingly, the compositional and antioxidant findings obtained in this study are interpreted within the scope of the five commercial samples analyzed. Despite the focused sample set, the integration of multi-element determination, spectroscopic and antioxidant characterization, and multidimensional analytical sustainability metrics provides a comprehensive analytical framework for the evaluation of traditional Turkish red sugar.

## 5. Conclusions

This study presents a holistic analytical approach to the characterization of five commercial traditional Turkish red sugar (loğusa/kızamık) samples, integrating multi-element determination with analytical performance and multidimensional sustainability assessment. Inductively coupled plasma mass spectrometer (ICP-MS) analyses performed using microwave-assisted extraction (MAE) and ultrasound-assisted deep eutectic solvent extraction (UA-DES-E) methods demonstrated that both procedures offer reliable calibration linearity, low detection limits, and satisfactory recovery values. Furthermore, the UA-DES-E method was shown to offer a significant advantage in terms of environmental burden due to its low-toxicity solvent components, avoidance of concentrated mineral acids, and relatively lower energy requirements. The tunable chemical structure and matrix-compatible extraction capacity of deep eutectic solvents offer significant potential not only in terms of sustainability but also in terms of selectivity and analytical efficiency. In this respect, these findings support the potential of DES-based extraction as a more sustainable sample-preparation alternative for multi-element analysis of this food matrix. One of the most important contributions of the study is that the evaluation of the methods is not limited to analytical performance parameters alone; the study involved the simultaneous application of multiple analytical metrics such as NEMI, AGREE, ComplexGAPI, AGSA, BAGI, CACI, VIGI, and RAPI. In today’s analytical method development studies, the claim of “greenness” has become a scientific necessity, requiring not only qualitative statements but also quantitative and multidimensional metrics. This multi-criteria evaluation provided a holistic comparison of methods in terms of solvent consumption, energy requirements, waste generation, safety profile, and overall analytical efficiency. The results show that the UA-DES-E method exhibited a more advantageous profile in terms of sustainability indicators. These findings highlight the need to evaluate performance and environmental impact criteria together when selecting methods in analytical chemistry. In conclusion, this study demonstrates the potential of DES-based extraction as a sustainable alternative for multi-element characterization of traditional food products. The compositional and antioxidant findings reported here specifically reflect the five commercial samples analyzed in the present study. Within this defined sample scope, the combined analytical and multidimensional metric-based approach provides a useful framework for the characterization and sustainability-oriented evaluation of traditional food matrices.

## Figures and Tables

**Table 1:** Operating Conditions of the ICP-MS.

Parameter	Data
Instrument	Agilent 7700 Series ICP-MS
RF Power (W)	1550
Plasma mode	Low matrix
Carrier gas	Argon
Carrier gas flow rate (L/min)	0.99
Plasma gas flow rate (L/min)	15
He gas flow rate (mL/min)	4.5
Spray Chamber Temperature (°C)	2
Nebulizer pump rate (rps)	0.3
Sample uptake time (s)	42
Stabilization time (s)	45
Integration time (s/point)	0.1–1
Tune mode	He/No gas
He mode isotopes	^44^Ca, ^24^Mg, ^39^K, ^55^Mn, ^56^Fe, ^63^Cu, ^52^Cr
No gas mode isotopes	^111^Cd, ^208^Pb
Software	MassHunter B.01.03

**Table 2:** Analytical performance characteristics for ICP-MS determination of selected elements.

Element	Slope (×10^3^)	Intercept (×10^3^)	R^2^	LOD (µg/kg)	LOQ (µg/kg)	Precision (%)		Recovery (%)	
				Inter-Day	Intra-Day	MAE	UA-DES-E		
^44^Ca	66.67	2.67	0.999	0.029 *	0.096 *	5.78	0.05	98.6	103.2
^24^Mg	51.37	3.62	0.999	0.028 *	0.093 *	2.59	0.18	97.2	103.8
^39^K	592.3	106.6	0.999	0.020 *	0.067 *	0.81	3.77	104.3	98.5
^63^Cu	6.83	1.36	0.999	0.565	1.883	0.91	0.05	103.7	98.7
^56^Fe	5228.9	10.50	1.000	2.288	7.628	2.15	0.08	104.8	96.2
^55^Mn	3.89	0.26	0.999	0.226	0.754	4.68	0.46	97.1	96.3
^52^Cr	5.77	0.49	0.999	0.172	0.576	0.85	0.34	104.1	101.2
^111^Cd	4.53	0.22	0.999	0.197	0.659	0.31	1.20	101.6	99.5
^208^Pb	37.99	5.97	0.999	0.659	2.197	3.37	0.04	105.1	104.5

* mg/kg.

**Table 3:** Moisture content of the analyzed samples.

Sample	Moisture (%)
S1	2.24 ± 0.05
S2	2.3 ± 0.08
S3	2.09 ± 0.04
S4	4.69 ± 0.22
S5	4.62 ± 0.10

**Table 4:** DPPH Free Radical Scavenging Activity of Samples.

Sample	DPPH Scavenging Effect (%)
S1	23.5 ± 0.9
S2	22.7 ± 1.4
S3	20.1 ± 2.7
S4	21.3 ± 3.9
S5	19.5 ± 0.3

Values are expressed as mean ± SD of triplicate measurements.

**Table 5:** Element concentrations obtained by MAE and UA-DES-E methods in the analyzed samples and paired *t*-Test results.

	Concentration (Mean ± SD)										Calculated *t*-Value
	MAE					UA-DES-E					
Element	S1	S2	S3	S4	S5	S1	S2	S3	S4	S5	
Ca (mg/kg)	86.2 ± 9.6	˂LOQ	21.1 ± 1.1	49.8 ± 4.2	19.1 ± 0.2	96.6 ± 5.8	<LOQ	20.1 ± 0.5	52.2 ± 3.5	19.5 ± 2.6	1.20
Cd (μg/kg)	˂LOQ	˂LOQ	˂LOQ	˂LOQ	˂LOQ	˂LOQ	˂LOQ	˂LOQ	˂LOQ	˂LOQ	-
Cr (μg/kg)	˂LOQ	4.63 ± 0.01	38.9 ± 0.34	221.1 ± 0.5	42.6 ± 0.19	<LOQ	4.46 ± 0.12	33.6 ± 0.17	227.1 ± 1.34	42.7 ± 1.62	0.07
Cu (mg/kg)	6.38 ± 0.96	5.18 ± 0.56	5.67 ± 0.63	3.71 ± 0.54	0.56 ± 0.45	5.1 ± 0.80	5.6 ± 1.38	6.2 ± 1.85	3.3 ± 1.70	0.8 ± 0.49	0.30
Fe (mg/kg)	7.94 ± 0.95	2.55 ± 0.90	5.78 ± 0.17	4.52 ± 0.96	2.03 ± 0.94	8.5 ± 0.76	2.9 ± 0.04	5.9 ± 0.32	4.0 ± 0.09	3.1 ± 0.21	1.21
K (mg/kg)	24.9 ± 0.39	39.1 ± 3.81	50.3 ± 5.72	52.5 ± 1.22	41.8 ± 0.35	22.9 ± 3.58	34.9 ± 1.93	58.5 ± 3.36	55.4 ± 3.22	42.1 ± 0.14	0.48
Mg (mg/kg)	17.6 ± 3.22	0.33 ± 0.02	16.4 ± 0.94	18.3 ± 0.87	8.1 ± 0.92	10.4 ± 0.87	0.23 ± 0.01	15.3 ± 0.96	17.8 ± 0.64	9.1 ± 0.36	1.10
Mn (μg/kg)	453.2 ± 1.3	386.3 ± 6.5	770.1 ± 4.5	242.8 ± 9.6	76.8 ± 8.7	427.8 ± 9.59	323.9 ± 3.97	744.6 ± 4.86	213.5 ± 7.12	74.1 ± 6.86	3.03
Pb (μg/kg)	426.1 ± 8.6	278.3 ± 4.2	259.6 ± 7.3	226.6 ± 2.2	12.1 ± 0.63	918.6 ± 6.66	180.5 ± 7.23	844.9 ± 8.51	695.9 ± 8.44	229.7 ± 8.53	2.69

Values are expressed as mean ± SD of three analytical replicates for each individual sample. S1–S5 represent five independent commercial samples (*n* = 5) used for the paired statistical comparison. For *n* = 5, the critical t-value was 2.776 at α = 0.05 (df = 4). For Ca and Cr, values below the LOQ were excluded from the paired *t*-test; therefore, the calculations were based on *n* = 4, with a critical *t*-value of 3.182 at α = 0.05 (df = 3).

**Table 6:** Comparative evaluation of UA-DES-E and MAE methods using sustainability and analytical assessment indexes (NEMI, ComplexGAPI, AGREE, AGSA, BAGI, CACI, and VIGI).

	UA-DES-E	MAE
NEMI	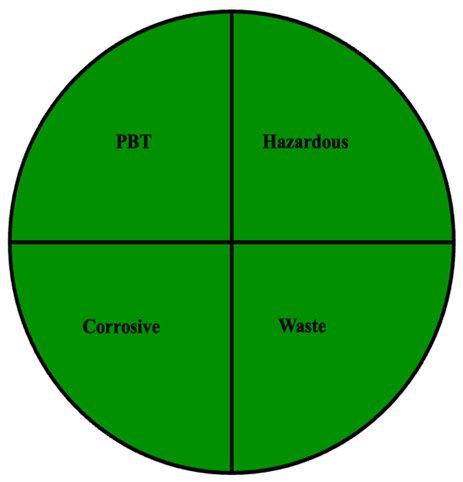	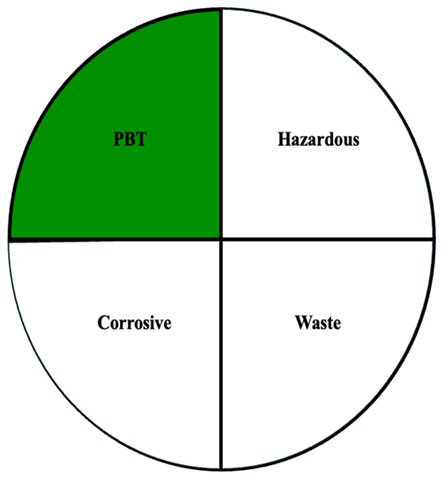
ComplexGAPI	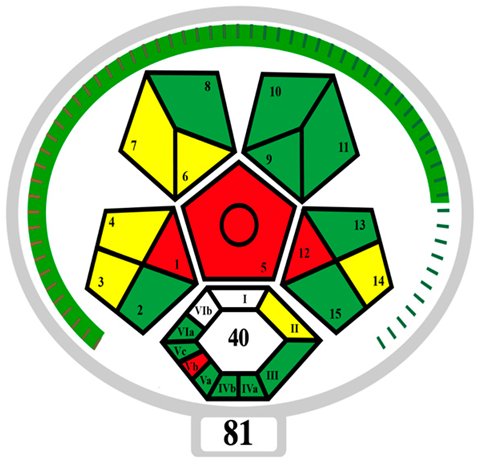	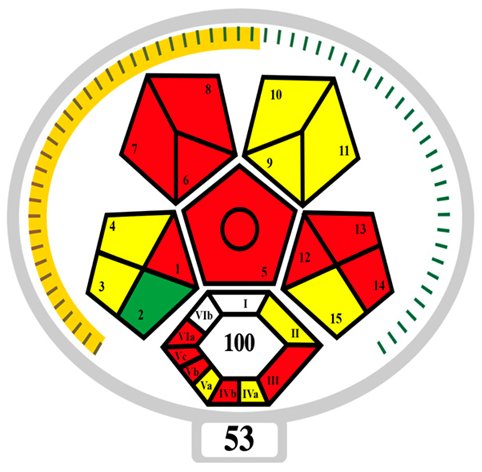
AGREE	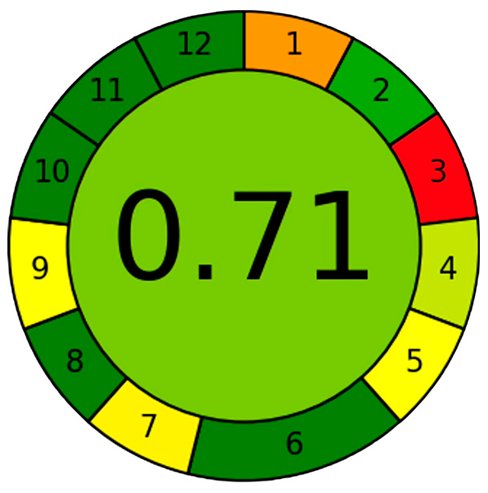	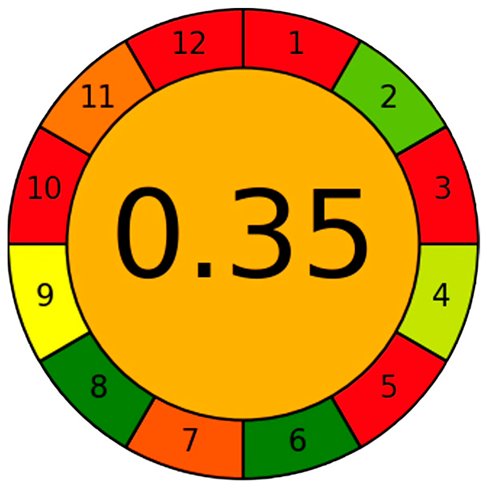
AGSA	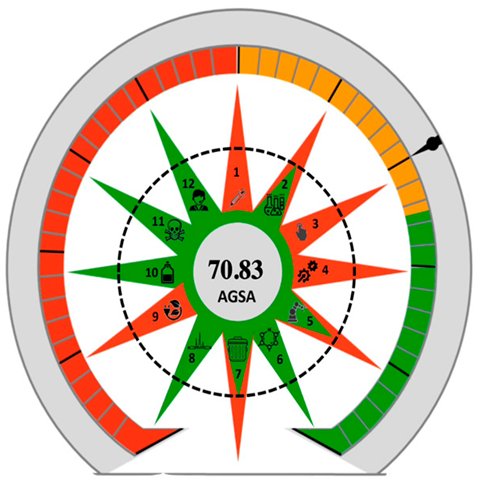	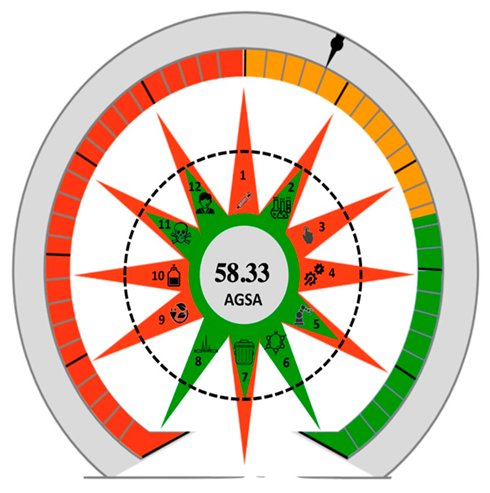
BAGI	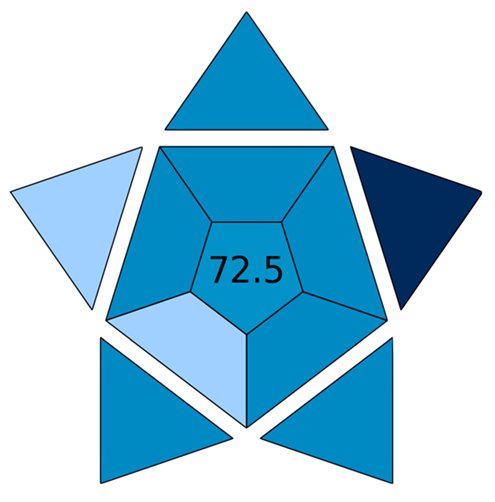	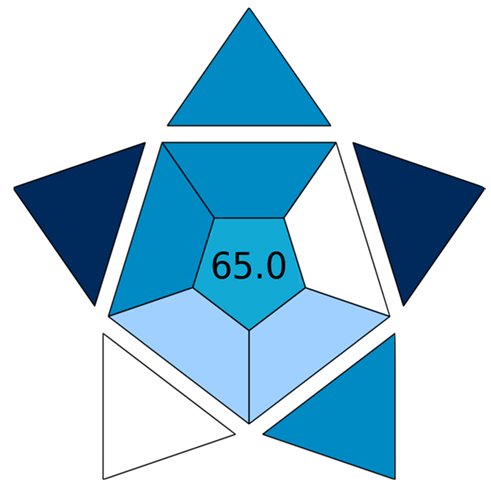
CACI	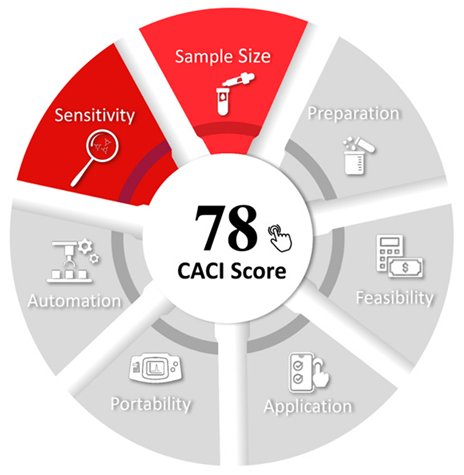	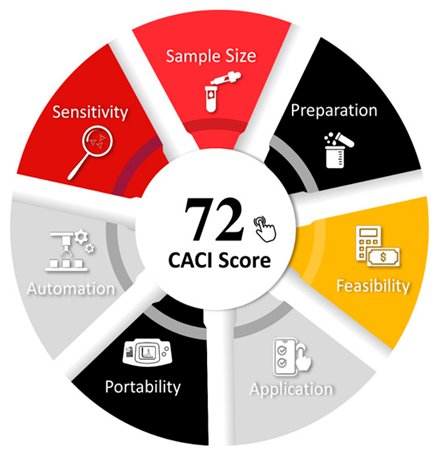
VIGI	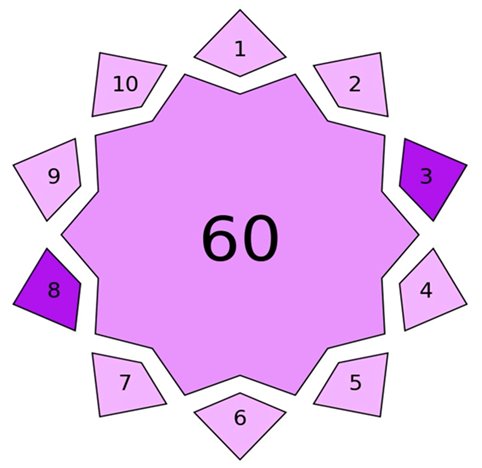	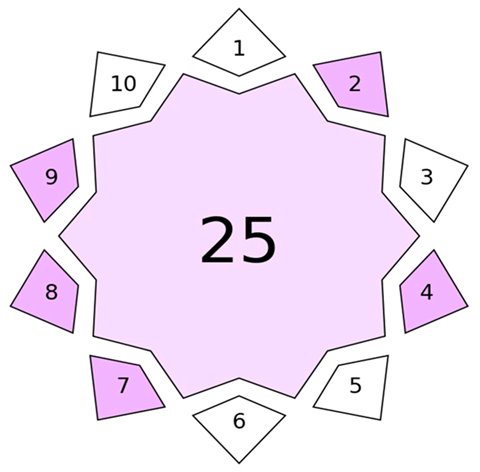

## Data Availability

The data supporting the findings of this study are available from the author upon reasonable request.

## Supplementary Materials

The following supporting information can be downloaded at: https://www.mdpi.com/article/10.3390/foods15203719/s1. Figure S1: Supporting spectroscopic information; Table S1: Input basis and scoring framework used for the multidimensional assessment of MAE and UA-DES-E.
