# Supplementary material for: Multi-Element Characterization and Sustainability Assessment of Traditional Turkish Red Sugar Using Deep Eutectic Solvent Extraction and ICP-MS
Source: Foods. 2026 Oct 19;15(20):3719. doi: 10.3390/foods15203719 (PMC13704269; doi:10.3390/foods15203719)
Supplement: Supplementary file 1 [file foods-15-03719-s001.zip › foods-4611327-supplementary.pdf]

Figure S1: Supporting spectroscopic information

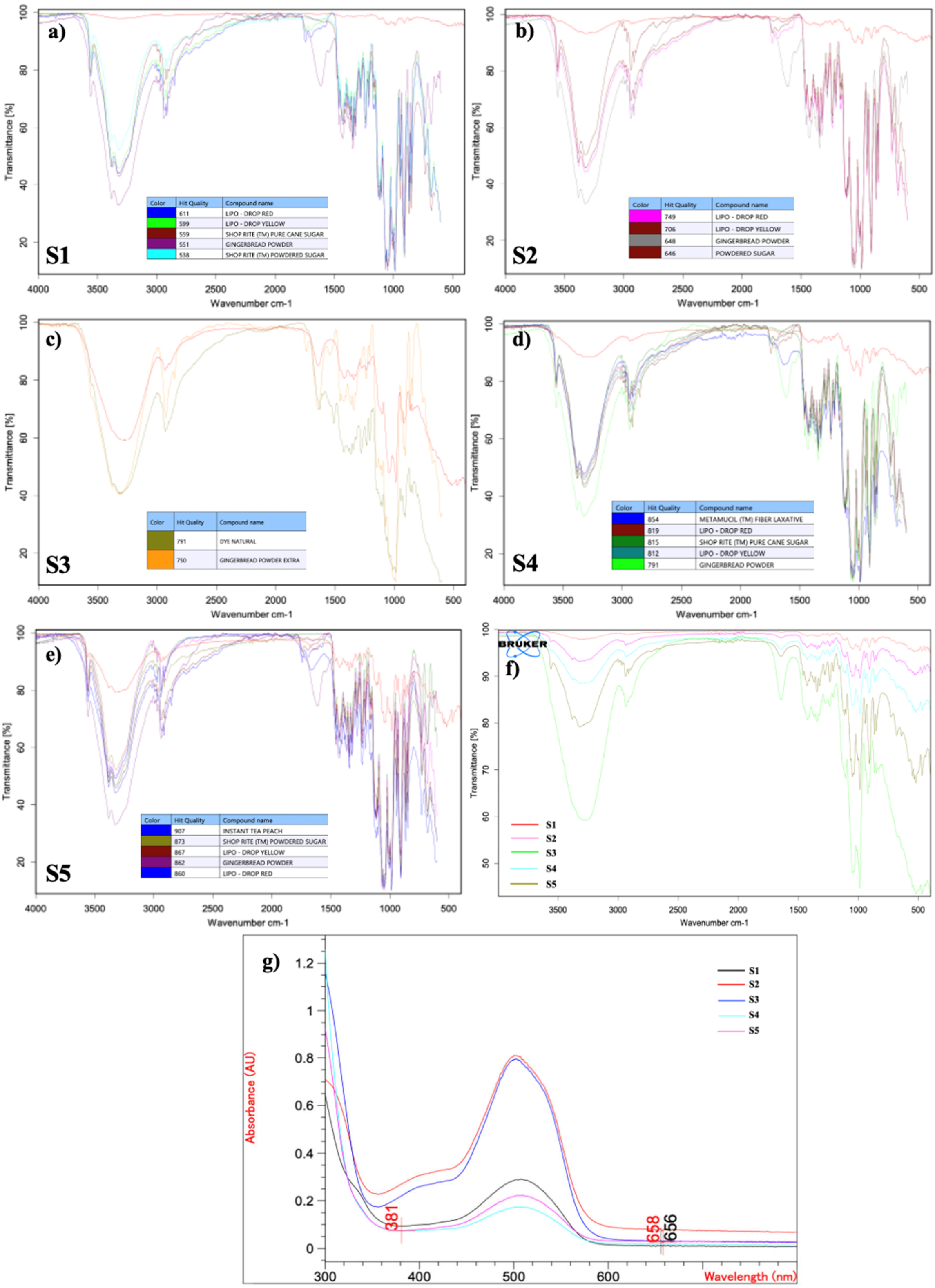

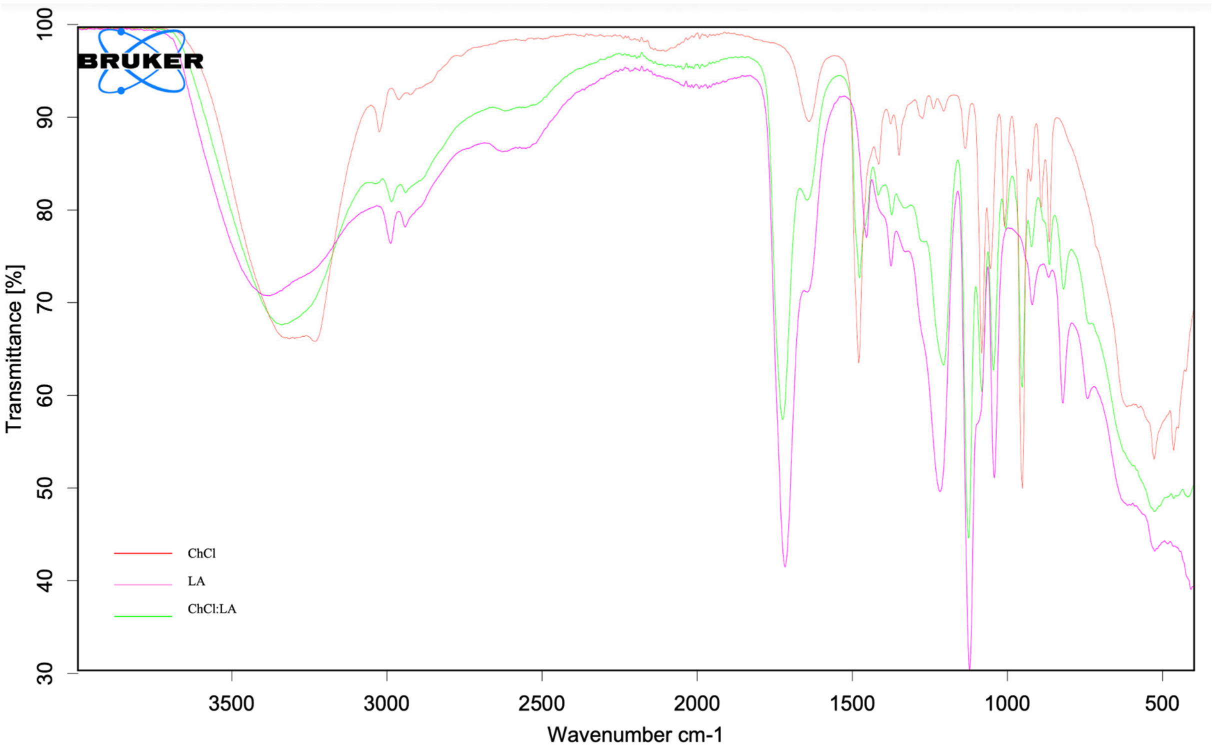

BRUKER
Transmittance [%]
100
90
80
70
60
50
40
30
3500
3000
2500
2000
1500
1000
500
Wavenumber cm-1
ChCl
LA
ChCl:LA

Table S1: Input basis and scoring framework used for the multidimensional assessment of MAE and UA-DES-E

Metric	Primary assessment dimension	Input/scoring basis used for MAE	Input/scoring basis used for UA-DES-E	Assessment logic
NEMI	Environmental hazard screening	Use of concentrated HNO_3 and H_2O_2; hazardous/corrosive reagent characteristics; waste generation	ChCl–lactic acid DES as the principal extraction medium; reduced hazardous-reagent use; waste generation	Four NEMI quadrants were evaluated according to PBT status, hazardous characteristics, corrosiveness, and waste generation.
AGREE	Environmental sustainability according to the 12 GAC principles	0.50 g sample; acid-assisted microwave digestion; HNO_3/H_2O_2 consumption; heating to 200 °C; microwave energy; multielement ICP-MS determination; no derivatization	0.25 g sample; 5 mL ChClacid-based DES; ultrasound-assisted extraction at 50 °C; reduced hazardous-reagent use; multielement ICP-MS determination; no derivatization	The analytical procedure was evaluated according to the 12 principles of Green Analytical Chemistry, including sample amount, sample preparation, reagent use, energy demand, waste, multianalyte capability, and operator safety.
ComplexGAPI	Environmental impact of the complete analytical workflow	Sample collection/storage; 0.50 g sample; HNO_3/H_2O_2 digestion; microwave treatment; dilution; ICP-MS analysis; reagent hazards, energy use, and generated waste	Sample collection/storage; 0.25 g sample; preparation and use of ChCl acid DES; ultrasound treatment; centrifugation, filtration and dilution; ICP-MS analysis	The complete analytical pathway, including pre-analysis processes, sample preparation, reagent/material use, instrumentation, energy requirements, and final determination, was considered.
AGSA	Quantitative greenness assessment	Sample amount; pretreatment; concentrated-acid consumption; microwave energy; hazardous waste; operator-safety considerations; no	Sample amount; DES-based extraction; ultrasound energy; reduced hazardous-reagent consumption; operator-safety	Method parameters were evaluated within the AGSA framework to provide a quantitative and visually interpretable greenness assessment.

		derivatization; multielement determination	considerations; no derivatization; multielement determination	
BAGI	Practical applicability	Quantitative multielement analysis; ICP-MS instrumentation; microwave-assisted sample preparation; commercially available reagents; sample amount; sample throughput; no preconcentration; degree of automation	Quantitative multielement analysis; ICP-MS instrumentation; ultrasound-assisted DES extraction; DES preparation; sample amount; sample throughput; no preconcentration; degree of automation	Ten BAGI attributes covering analytical determination, sample preparation, throughput, reagents/materials, preconcentration, automation, and sample amount were evaluated.
CACI	Operational simplicity and practicality	Sample preparation requirements; sample amount; microwave digestion; reagent requirements; ICP-MS applicability; analytical sensitivity; operational characteristics and automation	Sample preparation requirements; sample amount; ultrasound-assisted extraction; DES preparation; ICP-MS applicability; analytical sensitivity; operational characteristics and automation	The procedures were assessed according to the CACI framework with emphasis on simplicity, efficiency, feasibility, applicability, and operational characteristics.
VIGI	Methodological innovation	Microwave-assisted digestion coupled with ICP-MS; conventional acid digestion chemistry; analytical metrics; instrumentation; miniaturization/automation characteristics; methodological approach	DES-based ultrasound-assisted extraction coupled with ICP-MS; alternative solvent design; analytical metrics; reduced hazardous-reagent use; miniaturized sample preparation; methodological approach	Ten innovation attributes were evaluated, including sample preparation/instrumentation, data processing, analytical-chemistry metrics, materials/reagents, miniaturization, automation, interdisciplinarity, sensitivity, and novelty of the analytical approach.
RAPI	Analytical performance	Calibration linearity; LOD/LOQ; recovery; intra-/inter-day precision, and available validation characteristics	Calibration linearity; LOD/LOQ; recovery; intra-/inter-day precision;	Analytical-performance characteristics were assessed using the RAPI framework; parameters not experimentally investigated were not

			working range and available validation characteristics	treated as demonstrated validation characteristics.
